# High Density Lipoprotein (HDL) Promotes Glucose Uptake in Adipocytes and Glycogen Synthesis in Muscle Cells

**DOI:** 10.1371/journal.pone.0023556

**Published:** 2011-08-19

**Authors:** Qichun Zhang, Yun Zhang, Haihua Feng, Rui Guo, Lai Jin, Rong Wan, Lina Wang, Cheng Chen, Shengnan Li

**Affiliations:** 1 Jiangsu Provincial Key Lab of Cardiovascular Diseases and Molecular Intervention, Department of Pharmacology, Nanjing Medical University, Nanjing, People's Republic of China; 2 School of Pharmacy, Nanjing University of Chinese Medicine, Nanjing, People's Republic of China; Pennington Biomedical Research Center, United States of America

## Abstract

**Background:**

High density lipoprotein (HDL) was reported to decrease plasma glucose and promote insulin secretion in type 2 diabetes patients. This investigation was designed to determine the effects and mechanisms of HDL on glucose uptake in adipocytes and glycogen synthesis in muscle cells.

**Methods and Results:**

Actions of HDL on glucose uptake and GLUT4 translocation were assessed with 1-[^3^H]-2-deoxyglucose and plasma membrane lawn, respectively, in 3T3-L1 adipocytes. Glycogen analysis was performed with amyloglucosidase and glucose oxidase-peroxidase methods in normal and palmitate-treated L6 cells. Small interfering RNA was used to observe role of scavenger receptor type I (SR-BI) in glucose uptake of HDL. Corresponding signaling molecules were detected by immunoblotting. HDL stimulated glucose uptake in a time- and concentration-dependent manner in 3T3-L1 adipocytes. GLUT4 translocation was significantly increased by HDL. Glycogen deposition got enhanced in L6 muscle cells paralleling with elevated glycogen synthase kinase3 (GSK3) phosphorylation. Meanwhile, increased phosphorylations of Akt-Ser473 and AMP activated protein kinase (AMPK) α were detected in 3T3-L1 adipocytes. Glucose uptake and Akt-Ser473 activation but not AMPK-α were diminished in SR-BI knock-down 3T3-L1 cells.

**Conclusions:**

HDL stimulates glucose uptake in 3T3-L1 adipocytes through enhancing GLUT4 translocation by mechanisms involving PI3K/Akt via SR-BI and AMPK signaling pathways, and increases glycogen deposition in L6 muscle cells through promoting GSK3 phosphorylation.

## Introduction

Lower plasma levels and dysfunction of high density lipoprotein (HDL) are closely associated with metabolic syndrome including cardiovascular diseases, obesity, dyslipidemia and type 2 diabetes mellitus [1]. ABCA1 and ABCG1 are severely decreased in type 2 diabetes mellitus, which inhibit HDL formation and mature due to lower efflux of cholesterol and phospholipid from peripheral tissues [2], [3]. Scavenger receptor type I (SR-BI) that mediates HDL uptake is highly expressed under elevated glucose circumstance [4]. Furthermore, other proteins regulating HDL metabolism such as cholesterol ester transfer protein, lecithin cholesterol acyltransferase, lipoprotein lipase are also involved in regulation of HDL in type 2 diabetes mellitus [5], [6], [7]. Accumulated evidence suggests that HDL enhancement play a beneficial role in maintaining glucose homeostasis via insulin-dependent and -independent pathways in type 2 diabetes mellitus. For insulin-dependent mechanism, HDL reverses failure of oxidized LDL-induced beta cells and counters apoptosis of LDL- and VLDL-stimulated beta cells [8], [9]. Moreover, HDL and apolipoprotein AI (apoAI) promote glucose uptake and activate AMPK α in primary human skeletal muscle cells by an insulin-independent way [10], [11]. Meanwhile, oxidation metabolism is increased through phosphorylation of acetyl-CoA carboxylase β in skeletal muscle following the treatment of HDL [10].

Upon binding to SR-BI, HDL activates tyrosine kinase followed by activation of phosphoinositide 3-kinase (PI3K), mitogen-activated protein kinase pathways and Akt-phosphorylated endothelial nitric oxide synthase (eNOS) of endothelial cells [12]. Both PI3K/Akt and AMPK signaling pathways positively evoke glucose transporter 4 (GLUT4) exocytosis in the state of insulin stimulation and various stress status such as exercise and hypoxia [13], [14], [15]. GLUT4 is already identified as the most important type of glucose transporters in maintaining glucose homeostasis. Increased translocation of GLUT4 during continue recycle process between intracellular storage organelles and plasma membrane with insulin stimulation is closely associated with enhanced glucose uptake of adipocytes and skeletal muscle cells. Whether GLUT4 is involved in the HDL-regulated glucose metabolism is poorly depicted.

Net number of GLUT4 on cell surface is the consequence of exocytosis coupled with endocytosis. Endocytotic process occurs via clathrin- and/or caveolin-mediated manners. GLUT4 endocytosis is inhibited by clathrin-coated pits disruption [16]. Moreover, the internalization of GLUT4 is also associated with lipid raft microdomains of calveolin-riched in plasma membrane, and prevented by cholesterol depletion with agents such as methyl-β-cyclodextrin and cholesterol oxidase [17], [18]. Cholesterol depletion influences caveolae and clathrin-coated pits structure and function, and inhibition of GLUT4 endocytosis is reversed by cholesterol replenishment [17]. Meanwhile, rapid and specific cholesterol depletion of HDL from caveolae is observed [19]. However, direct evidence is required to reveal the role of reverse cholesterol transport, a typical function of HDL, in endocytotic process.

Following glucose uptake, glycogen synthesis is one of glucotropic effects of insulin. Glycogen synthase kinase-3 (GSK3) inhibits glycogen synthesis through glycogen synthase (GS) phosphorylation, which disrupts glucose homeostasis and favors insulin resistant status [20]. GSK3β activation is attenuated by phosphorylation following insulin-stimulated Akt phosphorylation [21]. Inactivation of GSK3β leads to GS dephosphorylation and glycogen synthesis inhibition in adipose, muscle and liver. Glycogen storage reduction in skeletal muscle is the phenotype of type 2 diabetes patients. Therefore, GSK3β, one of isoforms of GSK3, is an important enzyme in regulating glucose metabolism and acts as a key target in treatment of type 2 diabetes mellitus.

In the present study, we investigate the influences of HDL on glucose uptake and GLUT4 translocation in 3T3-L1 adipocytes and glycogen synthesis in L6 cells to provide more evidences for HDL in regulating glucose homeostasis. The results indicate that HDL promotes glucose uptake in adipocytes via enhancement of GLUT4 translocation that may be through SR-BI, and increases glycogen deposition in skeletal muscle cell following phosphorylation of GSK3.

## Results

### HDL Stimulates Glucose Uptake in 3T3-L1 Adipocytes

**Figure 1:**
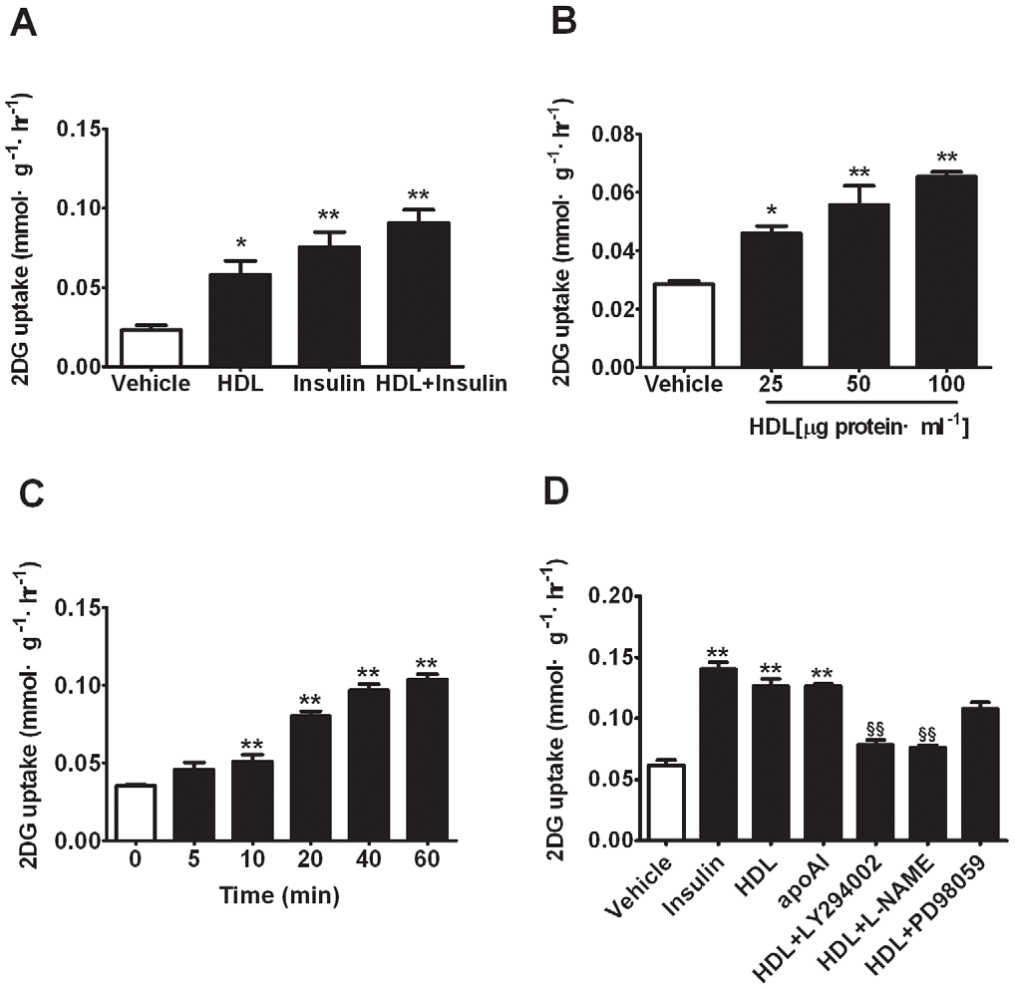
HDL stimulates glucose uptake in 3T3-L1 adipocytes. 3T3-L1 adipocytes were incubated with vehicle or stimulators for 30 min before being incubated for another 30 min with 1-[^3^H]-2- deoxyglucose. (A) 3T3-L1 adipocytes were incubated with vehicle (BSA), HDL (50 µg protein/ml), insulin (100 nmol/L) or indicated combination. (B) 3T3-L1 adipocytes were incubated with vehicle and HDL of indicated concentrations. (C) 3T3-L1 adipocytes were incubated with vehicle, HDL (50 µg protein/ml) at indicated time points. (D) 3T3-L1 adipocytes were incubated with vehicle, HDL (50 µg protein/ml), apoAI (50 µg/ml) and indicated combination with LY294002 (2 µmol/L), L-NAME (2 mmol/L) and PD98059 (1 µmol/L). Data are expressed as means±SEM of three independent experiments, n = 6. * p<0.05, ** p<0.01, *vs* vehicle; ## p<0.01, *vs* HDL.

To investigate the effect of HDL on glucose uptake, 1-[^3^H]-2-deoxyglucose uptake was directly assessed in 3T3-L1 adipocytes incubated with HDL for 30 min. HDL increased glucose uptake of 3T3-L1 adipocytes in a concentration-dependent fashion with significant effect at the range from 25 to 100 µg protein/ml (Figure 1A and 1B), consistent with previous report [10]. Time-dependent effect of HDL on glucose uptake was obtained during a period of 60 min, and dramatic increase of glucose uptake was observed at 20 min and no further enhancement was revealed (Figure 1C). To further investigate which pathways might be involved in the process of glucose uptake stimulated by HDL, cells were treated with HDL combined with LY294002, L-NAME and PD98059, respectively. Both LY294002 and L-NAME significantly blocked glucose uptake initiated by HDL, but no pronounced blockade of PD98059 was detected (Figure 1D). Meanwhile, similar enhancement in glucose uptake was simultaneously observed with apoAI incubation. These results show that HDL obviously stimulate glucose uptake through PI3K/Akt and eNOS-associated pathways. ApoAI may be the major component to mediate this action.

### HDL Increases Glycogen Synthesis in L6 Cells

**Figure 2:**
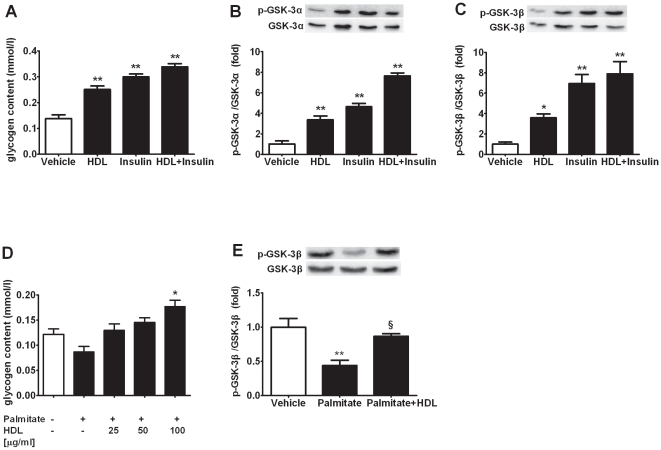
HDL increases glycogen synthesis in L6 cells and muscle tissue in vivo. (A) L6 cells starved overnight were incubated with vehicle, HDL (50 µg protein/ml), insulin (100 nmol/L) or indicated combination for 2 h, n = 6. (B and C) GSK3α (B) and GSK3β (C) phosphorylation in lysates from incubated L6 cells at basal condition and after incubation with stimulators as (A), n = 3. (D) L6 cells were treated with palmitate (0.4 mmol/L) for 12 h followed by incubation with HDL of indicated concentrations for 2 h, n = 6. (E) GSK3β phosphorylation in lysates from incubated L6 cells under conditions as (D), n = 3. Data are expressed as means±SEM of three independent experiments performed in vitro. * p<0.05, ** p<0.01, *vs* vehicle; § p<0.05, *vs* palmitate.

To further investigate whether glycogen synthesis was modulated by HDL, glycogen content was determined as glycosyl units with amyloglucosidase. As the data indicated, glycogen contents markedly increased in L6 skeletal muscle cells after acute HDL exposure (Figure 2A). Our data also showed that HDL induced GSK3α and GSK3β phosphorylations in L6 muscle cells, contributing to the increase of glycogen synthesis (Figure 2B and 2C). Moreover, excessive free fatty acids (FFAs) were documented to induce insulin resistance and glycogen synthesis inhibition [22]. HDL treatment reversed the glycogen synthesis decrease and GSK3β activation caused by high levels of palmitate in L6 cells (Figure 2D and 2E). These results indicate that HDL enhances the deposition of glycogen in muscle cells through phosphorylation of GSK3β.

### HDL Enhances GLUT4 Exocytosis and Inhibits GLUT4 Endocytosis in 3T3-L1 Adipocytes

**Figure 3:**
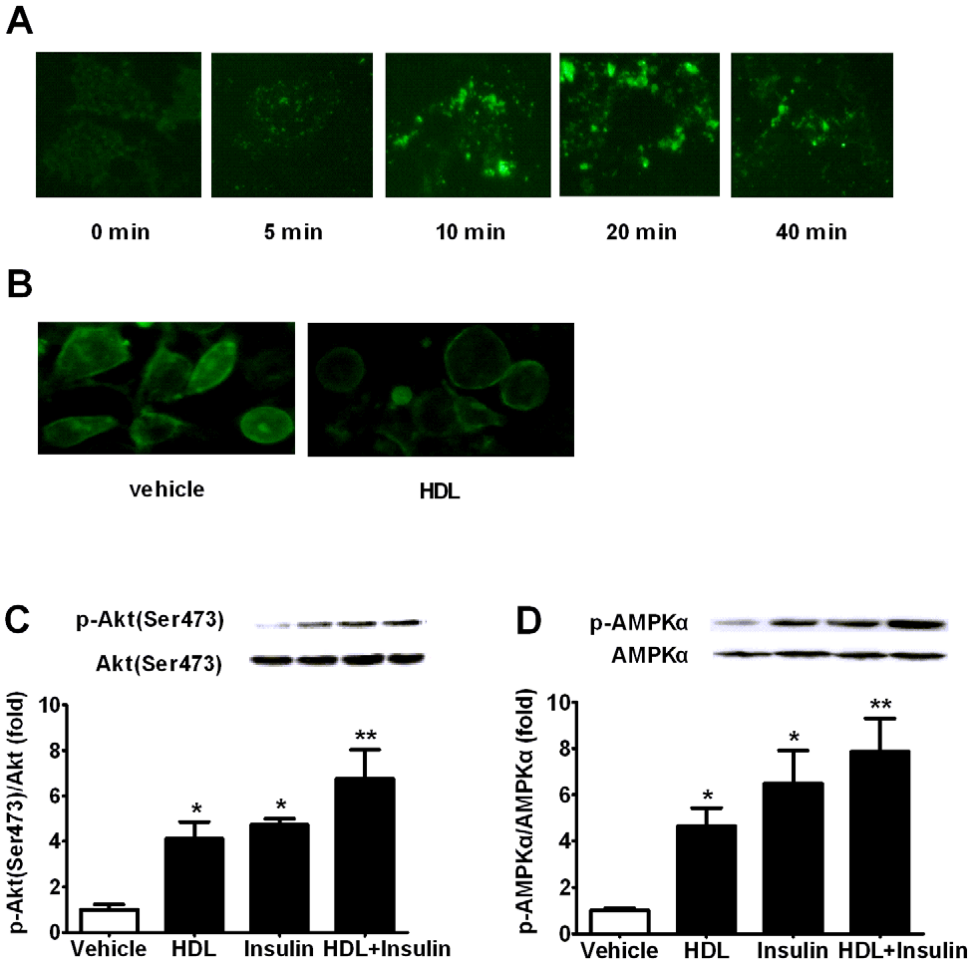
HDL enhances GLUT4 exocytosis and inhibits GLUT4 endocytosis in 3T3-L1 adipocytes. (A) 3T3-L1 adipocytes were treated in the absence and presence of 50 µg protein/ml HDL for indicated time points. GLUT4 was immunofluorescently labeled with GLUT4 antibody and followed by FITC-conjugated goat anti-rabbit IgG incubation in plasma membrane sheets. (B) 3T3-L1 adipocytes were chilled to 4°C, and labeled with FITC-cholera toxin B. Then adipocytes were incubated in the absence or presence of HDL for 2.5 h to allow cholera toxin B internalization. (C and D) Akt (C) and AMPKα (D) phosphorylation in lysates from 3T3-L1 adipocytes incubated with vehicle and indicated stimulators analyzed with corresponding antibodies. Data are expressed as mean±SEM of three independent experiments. n = 3. * p<0.05, ** p<0.01, *vs* vehicle.

We subsequently investigated whether GLUT4 participated in the regulation of glucose utilization by HDL. Incubation of 3T3-L1 adipocytes with 50 µg protein/ml HDL increased GLUT4 exocytosis in a time-dependent manner and the maximal fluorescence, representing GLUT4 amount, was obtained at around 20 min after HDL exposure (Figure 3A), which was in accordance with time-effect course of HDL on glucose uptake in 3T3-L1 adipocytes (Figure 1B). On the other hand, more fluorescence was observed on membrane of HDL-treated cells after incubation with FITC-CTB, which indicated a marked reduction of CTB endocytosis (Figure 3B). CTB was commonly employed to evaluate GLUT4 endocytosis via caveolin-dependent mechanism. This result suggests that GLUT4 endocytosis may also be inhibited by HDL. Since activations of signaling PI3K/Akt and AMPK initiate glucose uptake through promoting GLUT4 translocation, phosphorylations of Akt and AMPKα were subsequently detected. A significant elevation in both Akt and AMPKα phosphorylations was observed after HDL exposure (Figure 3C, 3D), which is consistent with HDL function in endothelial [23], [24]. These findings indicate that GLUT4 amount in plasma membrane may increase with enhanced exocytosis through Akt & AMPK pathways and delayed endocytosis mediated by caveolin in the presence of HDL.

### HDL Prompts Glucose Uptake via SR-BI

**Figure 4:**
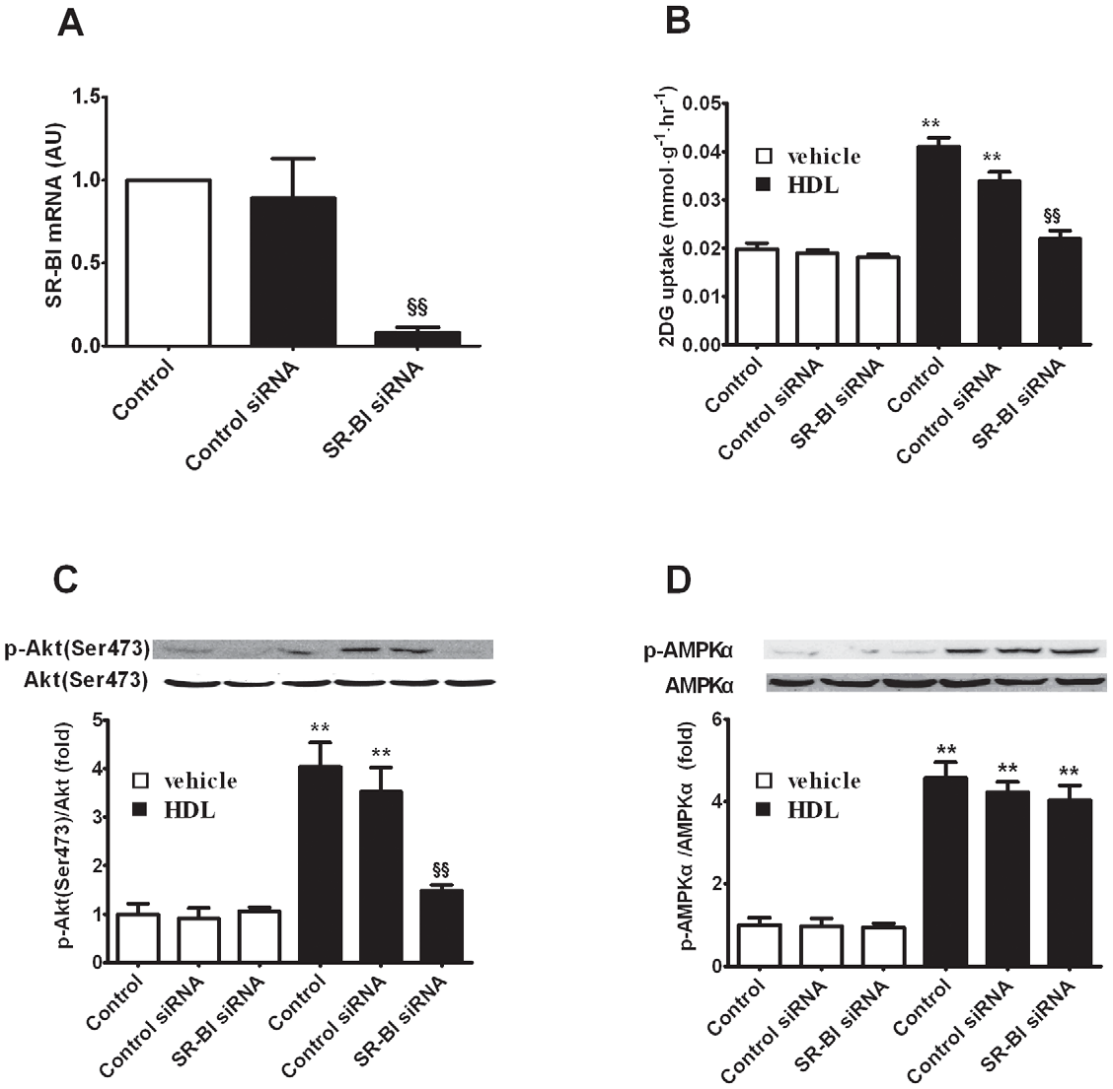
HDL prompts glucose uptake via SR-BI. (A) The relative expression of SR-BI to GAPDH in 3T3-L1 adipocytes with treatment of vehicle, control siRNA and SR-BI siRNA. (B) Glucose uptake in 3T3-L1 adipocytes with and without SR-BI RNA interfering were incubated in the absence or presence of HDL (50 µg protein/ml), n = 6. (C and D) Akt (C) and AMPKα (D) phosphorylation in 3T3-L1 adipocytes with and without SR-BI RNA interfering were incubated in the absence or presence of HDL (50 µg protein/ml), n = 3. Data are expressed as mean±SEM of three independent experiments. ** p<0.01, *vs* vehicle; §§ p<0.01, *vs* control RNA interfering.

In addition to mediating reverse cholesterol transport between cells and lipoprotein, SR-BI is also considered to realize cardiovascular protection of HDL through activating downstream signal molecules such as Akt, mitogen-activated protein kinase and AMPK [23]. The roles of Akt and AMPK in glucose metabolism prompt the hypothesis that SR-BI may directly mediate HDL functions in glucose metabolism aforementioned. Glucose uptake and phosphorylations of Akt and AMPKα were assessed in 3T3-L1 adipocytes of SR-BI knock-down with RNA interference (Figure 4A). Significant glucose uptake stimulated by HDL was diminished in SR-BI knock-down (Figure 4B). Consistent with this result, pronounced inhibition of Akt activation was observed with lower SR-BI levels (Figure 4C). Unexpectedly, SR-BI knock-down seemed to have no influence on AMPKα phosphorylation (Figure 4D). These results demonstrate that SR-BI is an important receptor for HDL in regulating glucose utilization.

## Discussion

In the present study, we characterized that HDL stimulated glucose uptake in 3T3-L1 adipocytes in concentration&time-dependent manners. Furthermore, the influence of HDL on glucose uptake was inhibited by LY294002 and L-NAME. It is well known that PI3K is the upstream of Akt and NOS. NOS activation could be modulated by HDL through Akt, which is initiated through the binding of HDL to SR-BI [12], [25], [26], [27]. Activation of PI3K/Akt and NOS promotes translocation of GLUT4 vesicle to plasma membrane [28], [29]. Therefore, it is reasonable to think that PI3K-Akt-NOS works as one pathway to regulate glucose uptake. Meanwhile, HDL prompted translocation of GLUT4 to plasma membrane from intracellular storage organelles. Any approach to expand retention of GLUT4 in plasma membrane would lead to enhancement of glucose uptake by peripheral tissues, especially skeletal muscle and adipose tissue. PI3K/Akt and AMPK signaling pathways have been showed to participate in GLUT4 exocytosis and activation [13], [14], [15]. GLUT4 translocation rather than its total amount variation plays a crucial role under diabetic conditions. The present study demonstrated that HDL prompted GLUT4 exocytosis in parallel with activating both PI3K/Akt and AMPK signaling pathways. Meanwhile, glucose transport in 3T3-L1 adipocytes with HDL stimulation was disturbed in the presence of LY294002, consistent with the results obtained in muscle cells infected with AMPK-DN virus [10]. Moreover, GLUT4 endocytosis followed by its dysfunction could be regulated through changing cholesterol contents in plasma membrane. Both Chromium and methyl-β-cyclodextrin are suggested to induce GLUT4 endocytosis coupled to membrane cholesterol loss that leads to insulin sensitizing in 3T3-L1 adipocytes [18]. Cholesterol efflux mediated by HDL is rapid and specific from lipid raft/caveolae, whereas that from non-raft microdomains is slow and lipoprotein independent [19]. Cholesterol depletion from caveolae influences its organization and structure, leading to internalization delay of GLUT4 mediated by caveolae [17], [30]. Furthermore, TC10, downstream of caveola/Cb1 signaling complex, has also been shown to be the second signal to GLUT4 exocytosis. Our result that caveolae-mediated CTB endocytosis was disturbed by HDL in 3T3-L1 adipocytes demonstrated HDL's inhibitory effect on GLUT4 endocytosis. Multiple functions of caveolae, especially abundant in adipocytes, have been discovered. The functions of caveolae include lipid regulation, membrane proteins endocytosis and mechanosensation [31]. Caveolin is the major membrane protein of caveolae. Caveolin-1 and -3, isoforms of caveolin, have a direct interaction with G proteins-coupled receptors, receptor tyrosine kinases, and so on. Some protein interactions within caveolae modulate cellular metabolism such as enhancement of insulin receptor pathway. The insulin signaling is augmented through Cb1-CAP complex and flotillin mediates translocation of the complex to caveolae followed by GLUT4-mediated glucose uptake [16]. Moreover, cholesterol depletion from caveolae results in change of structure and function, which ultimately interferes GLUT4 exocytosis and endocytosis processes. SR-BI, the receptor mediating cholesterol transport of HDL from peripheral tissues, is reported to colocalize with calovae [32]. There are three putative pathways to reasonably interpret the influence of HDL on glucose uptake via GLUT4 translocation. First, Activation of PI3K/Akt signaling pathway is achieved by binding with SR-BI receptor. Second, HDL influences the recruit of dimeric Cb1-CAP complex to flotillin in lipid raft through cholesterol depletion. Third, HDL inhibits endocytosis process of GLUT4 mediated by caveolin.

Besides cholesterol transport from peripheral tissues to liver and steroidogenic organs, SR-BI has been demonstrated to initiate diverse HDL-induced kinase cascades. SR-BI mediates cellular uptake of bioactive lipid such as S1P and its precursor sphingosine from HDL, which facilitates the binding of S1P to Gi-coupled S1P receptors and initiates following signaling activation [33]. On the other hand, SR-BI modulates cholesterol distribution in plasma membrane and influences the structure/morphology of membrane. Depletion of cholesterol conducted by SR-BI from caveolae inhibits assembly and activity of PPA2A/HePTP phosphatase complex, which subsequently results in decreased dephosphorylation of protein kinase targets such as ERK1/2 [34], [35]. Direct binding to SR-BI, HDL leads to activation of eNOS in endothelial cells, which is regulated by PI3K/Akt signaling pathway and mitogen activated protein kinases ERK1/2 pathway [12], [27]. Moreover, migration of endothelial cells and inhibition of adhesion molecules could also be regulated by the interaction of HDL and SR-BI. In the present study, using RNA interference, we provide evidence that SR-BI is involved in glucose uptake stimulated by HDL in 3T3-L1 adipocytes. The effect of HDL on glucose uptake is reversed by SR-BI knock-down. Activation of signaling molecule Akt is attenuated by the RNA interference of SR-BI. Although it is well documented that SR-BI plays important role in regulating glucose homeostasis by some indirect ways such as increasing insulin secretion and inhibiting pancreatic β-cells apoptosis [36], this work shows the direct effect of HDL on glucose uptake in adipocytes.

Glucose transported in skeletal muscle is undergone oxidative glucose disposal and/or synthesized into glycogen, which is dependent on energy requirement of skeletal muscle. Deficiencies of glycogen synthesis and storage in skeletal muscle are features of type 2 diabetes patients. Meanwhile, glucose metabolism in skeletal muscle is closely associated with enhanced plasma concentration of FFAs in individuals with type 2 diabetes. Elevated FFAs levels could reduce activity of hexokinase II, which decreases glucose uptake into skeletal muscle and subsequently inhibits nonoxidative glucose metabolism by inhibition of GS [37]. GSK3 is the downstream protein kinase of PI3K/Akt and AMPK signaling molecules and regulates GS activity. The recent report indicated that both HDL and apoAI increased glucose uptake and fatty acid oxidation by activating AMPK and the upstream CaMKK in skeletal muscle of type 2 diabetes subjects [10]. Our present study showed that HDL increased glycogen synthesis in L6 cells. Glycogen storage enhancement was achieved through GSK-3β phosphorylation. Higher FFAs were verified to decrease glycogen synthesis by GSK-3β dephosphorylation, impair pancreatic islet secretion and disturb insulin signal transduction [22]. Here, we found that decreased glycogen content and increased GSK-3β activation induced by palmitate in L6 muscle cells were attenuated by HDL incubation. HDL-induced higher glycogen levels in skeletal muscle could be completed by the direct regulation of GSK3β. Increase of fatty acid oxidation is also expected to involve in this result because elevated fatty acid oxidation inhibits glucose oxidation which strengthens glycogen synthesis.

In conclusion, the present investigation provides evidence that HDL promotes glucose uptake in adipocytes by regulating GLUT4 translocation and increases glycogen deposition in skeletal muscle cells. These findings provide new insights into the influences and mechanisms involved in glucose metabolism of HDL.

## Materials and Methods

### Cell Culture

Murine-derived 3T3-L1 fibroblasts (Chinese Academy of Sciences Committee Type Culture Collection cell bank, Shanghai)were cultured in DMEM with 25 mmol/l glucose, supplemented with 10% FBS, 100 µg/ml streptomycin and 100 U/ml penicillin, under an atmosphere of 95% air/5% CO_2_ at 37°C. 3T3-L1 fibroblasts were differentiated into adipocytes 2 days post confluent with the same DMEM medium containing 3-isobutyl-1-methylxanthine (0.5 mmol/l, Sigma), dexamethasone (1 µmol/l, Sigma), insulin (1 mg/l, Sigma) and 10% FBS. Induction medium was aspirated and replaced by medium supplemented with 10% FBS and insulin (1 mg/l) in the next 2 days. Thereafter, the cells were maintained for additional 4–10 days in DMEM with 10% FBS, and the medium was changed every other day. Adipocytes were identified with Oil-red (Sigma) staining, and those expressed adipocyte phenotype >90% were used. Before experiments, adipocytes were incubated in serum-free medium containing 0.2% bovine serum albumin (BSA) for starvation overnight.

Rat L6 skeletal muscle cells (Chinese Academy of Sciences Committee Type Culture Collection cell bank, Shanghai) were cultured in α-MEM supplemented with 10% FBS, 100 µg/ml streptomycin and 100 U/ml penicillin, in an atmosphere of 95% air/5% CO_2_ at 37°C. Cells were grown in 100 mm culture dish to confluence and then switched to α-MEM containing 2% FBS and differentiated for 12–14 days. Fully differentiated myotubes were starved in serum-free medium containing 0.5% BSA overnight prior to experiments.

### Glucose Transport

3T3-L1 adipocytes were washed with KRPH buffer (5 mM Na_2_HPO_4_, 20 mM HEPES, pH 7.4, 1 mM MgSO_4_, 1 mM CaCl_2_, 136 mM NaCl, 4.7 mM KCl, and 1% BSA) three times, incubated with KRPH for 1 h, and either treated or untreated as demonstrated in figure legends for specific times. Glucose transport was analyzed by incubation in KRPH containing 0.5 µCi 1-[^3^H]-2-deoxyglucose (GE Healthcare, Waukesha, WI) and 50 µmol/l 2-deoxyglucose in the absence or presence of 10 µmol/l cytochalasin B (Sigma-Aldrich, Inc., St. Louis, MO). The transport was stopped by rinsing the cells with ice-cold PBS for three times. The adipocytes were lysed in 1% Triton X-100 at 37°C for 30 min, and then the aliquots were subjected to scintillation counting.

### Plasma Membrane Sheet Assay

The translocation of GLUT4 was assessed by plasma membrane sheet assay [38]. 3T3-L1 fibroblasts were plated on poly-L-lysine-coated glass coverslips in six-well plates and then differentiated into adipocytes. For stimulation with acute HDL, adipocytes was starved in serum free DMEM containing 0.5% BSA for 18 h, and then washed twice with KRPH buffer (5 mmol/L Na_2_HPO4, 20 mmol/L HEPES pH 7.4, 1 mmol/L MgSO_4_, 1 mmol/L CaCl_2_, 136 mmol/L NaCl, 4.7 mmol/L KCl, and 1% BSA) followed by equilibration for 1 h at 37°C prior to the presence of HDL (50 µg protein/mL). After HDL incubation for 0, 5, 10, 20, 40 min, we collected the coverslips with 3T3-L1 adipocytes and rinsed them in ice-cold PBS. Subsequently, the cells were incubated with 3 mL poly-L-lysine (0.5 mg/mL) in PBS for 1 min, and swollen by three successive 5-s rinses in a 100 mm dash with 10 mL ice-cold hypotonic buffer (23 mmol/L KCl, 10 mmol/L HEPES pH 7.5, 2 mmol/L MgCl_2_, and 1 mmol/L EGTA) and immediately transferred to 10 mL ice-cold sonication buffer (70 mmol/L KCl, 30 mmol/L HEPES pH 7.5, 5 mmol/L MgCl_2_, 3 mmol/L EGTA, 1 mmol/L dithiothreitol, and 0.1 mmol/L phenylmethyl-sulfonyl fluoride), and sonicated to broken through placing a 5-mm sonication microprobe just touch the surface of the buffer. For immunofluorescence labeling, the plasma membrane lawn were rinsed two times with ice-cold sonication buffer and placed in a solution containing 2% paraformaldehyde, 70 mmol/l KCl, 30 mmol/l HEPES, pH 7.5, 5 mmol/l MgCl_2_, and 3 mmol/l EGTA for 20 min at room temperature. The fixed lawn washed in PBS for 5 s were blocked at 4°C overnight in PBS containing 5% skim dry milk and incubated overnight at 4°C with a 1∶100 dilution of polyclonal rabbit GLUT4 antibody, and followed by PBS rinse for three times and incubation with a 1∶10 dilution of fluorescein isothiocyanate (FITC)-conjugated goat anti-rabbit IgG for 60 min at room temperature. Fluorescence images were obtained using Olympus (Olympus, Japan) Fluorescence microscopy.

### Cholera Toxin B Uptake

The uptake of Cholera toxin B was determined as previous description to measure caveola-mediated endocytosis [17]. Briefly, 3T3L1 adipocytes were rinsed twice with 37°C Hanks' balanced salt solution (HBSS) and then incubated in serum-free DMEM containing 2% BSA and starved for 2 h. The cells were subsequently chilled to 4°C and incubated with 4 µg/ml FITC-cholera toxin B in serum-free DMEM containing 2% BSA. Unbound toxin-B was removed by rinsing labeled cells four times with chilled HBSS. Being treated, these labeled 3T3-L1 adipocytes were incubated 2.5 h in the same medium at 37°C to allow internalization and then fixed with 2% paraformaldehyde in HBSS. The internalization of FITC-cholera toxin B was monitored by fluorescence microscopy.

### Immunoblotting

To obtain total protein, adipose tissue, gastrocnemius muscle or indicated cells were lysised in RIPA buffer (500 mmol/l Tris-HCl pH 7.4, 1 mmol/l EDTA, 150 mmol/l NaCl, 1% NP-40, 0.25% Na-deoxycholate, 1 mmol/l phenylmethylsulfonl fluoride, and protease inhibitor cocktail) and then centrifuged (12,000 rpm) for 10 min at 4°C. The protein concentrations were determined using Lowry protein assay reagent. Protein samples were electrophoresed on a sodium dodecyl sulfate (SDS)- polyacrylamide gel after boiling for 5 min in SDS sample buffer. Proteins blots were transferred to luminescence membranes (Millipore). After electroblotting, the membranes were blocked with 5% skim dry milk in TBS and Tween 20 (10 mmol/l Tris-HCl pH 7.4, 150 mmol/l NaCl, and 0.1% Tween 20) and then incubated at 4°C with the corresponding primary antibody diluted in blocking buffer overnight. Membranes were incubated with the appropriate second antibodies for 1 h at room temperature. Immunoreactive bands were detected by the enhanced chemi-luminescence system. The density of bands was quantified using a Gel-pro analyzer.

### Glycogen Assay

Glycogen contents of L6 cells and muscle tissue were assayed as described previously [39]. Briefly, well differentiated L6 cells in 100 mm dish were digested with 1 N NaOH at 80°C for 15 min. Protein was then precipitated with an equal volume of 2 N trichloroacetic acid and centrifuged at 10,000 g for 15 min. Glycogen precipitation from the supernatant was achieved by using 100% ethanol (2∶1, v/v). The glycogen pellet was rinsed with 80% ethanol and reconstituted in water, and the conversion of glycogen to glucose was performed with 1 mg/ml amyloglucosidase (Sigma-Aldrich, Inc., St. Louis, MO) in 0.5 mol/l sodium acetate buffer, PH 4.8. The resulting glucose concentration was determined based on glucose oxidase-peroxidase enzyme system.

### Small Interfering RNA Transfection

To knock-down SR-BI in 3T3-L1 cells, the lentiviral pLKO.1 vector carrying the sequence GGCTATGACGATCCCTTCGTGCATT was employed to produce virus for interfering RNA of SR-BI [40]. 3T3-L1 cells were infected with lentiviral supernatants generated from 293T cells by transient cotransfection with lipofectAMINE2000 (Invitrogen, Carlsbad, CA). After 48 h, 3T3-L1 cells were selected with puromycin (2 mg/ml) for additional 14 d and used for subsequent experiments. As a control, pLKO.1 vector without insert was transfected simultaneously into separate cells under the same condition. RNA level was assayed with quantitative real-time RT-PCR in SR-BI knock-down cells.

### Real-time RT-PCR

Total mRNA was isolated from 3T3-L1 with TRIzol according to the manufacturer's instructions (Invitrogen). RNA then was reverse-transcribed with a TaqMan reverse transcription kit. Real-time PCR was performed using an ABI 7300 system (Applied Biosystems) under standard reaction conditions. SYBR Green oligonucleotides (Applied Biosystems) were used for detection and quantification of SR-BI mRNA levels, which were normalized to glyceraldehyde-3-phosphate dehydrogenase (*GAPDH*) using the ΔΔ*C*_T_ method (Figure 2). The specificity of the PCR amplification was verified by melting curve analysis of the final products. Primer sequences used to amplify *SR-BI* and *GAPDH* were as follows [41]: F: 5′-CTCCCAGACATGCTTCCCATA-3′ and R: 5′-GTCAGCTTCATGGACCTGCA-3′ for *SR-BI*; F: 5′-ATTCAACGGCACAGTCAAGG-3′ and R: 5′-TGGATGCAGGGATGATGTTC-3′ for *GAPDH*.

### Statistical Analysis

Results are expressed as means ± SE and analyzed using one-way ANOVA and Student's *t* test. A Newman Keuls *post hoc* analysis was conducted to pairwise multiple comparisons as significance was reached by ANOVA. The difference was considered to be statistically significant when *P<0.05*.
